# Double EUS-guided bypass for gastric outlet and biliary tract malignant obstruction: A standardized one-step approach (with videos)

**DOI:** 10.1097/eus.0000000000000075

**Published:** 2024-08-20

**Authors:** Victor Lira de Oliveira, Marcos Eduardo Lera dos Santos, Mateus Bond Boghossian, João Remí de Freitas Júnior, Maria Luíza Lemos Pires Pereira, Carolina Vaz Turiani, Eduardo Guimarães Hourneaux de Moura

**Affiliations:** 1 Gastrointestinal Endoscopy Unit—Gastroenterology Department, Hospital das Clínicas da Faculdade de Medicina da Universidade de São Paulo, SP, Brazil; 2 Gastrointestinal Surgery Unit—Hospital dos Servidores do Estado de Pernambuco, Recife, PE, Brazil; 3 Gastrointestinal Surgery Unit—Associação Beneficência Portuguesa de São Paulo, SP, Brazil.

Various malignant conditions, including gastric cancer, periampullary neoplasms, and pancreatobiliary tumors, can result in concurrent biliary tract and gastric outlet obstruction (GOO). Nowadays, less invasive approaches such as duodenal stenting and transpapillary drainage via endoscopic retrograde cholangiopancreatography are preferred over surgical treatment.^[1,2]^

EUS–guided therapies, specifically EUS-guided gastroenterostomy (EUS-GE) and EUS-guided biliary drainage (EUS-BD), are valuable alternatives. They offer higher success rates and fewer stent-related issues, particularly in challenging cases where traditional methods may exhibit decreased efficacy.^[3,4]^

Two video-documented cases showcase combined EUS-guided bypass procedures for gastric outlet obstruction (GOO) and biliary tract obstruction, illustrating the basic principles of the technique (Videos 1 and 2).

**Video Legend** Videos are only available at the official website of the journal (www.eusjournal.com).

**Video Legend**

**Figure 1:**
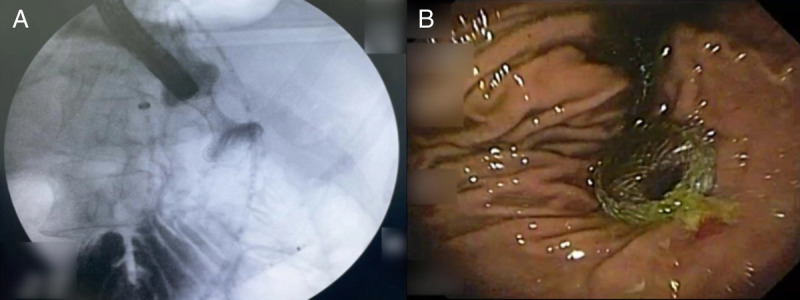
Intraprocedural images. A, Fluoroscopic confirmation of EUS-gastroenterostomy LAMS positioning with contrast injection. B, Endoscopic view of EUS-guided cholecystogastrostomy LAMS with satisfactory bile outflow. LAMS: Lumen-apposing metal stent.

A 64-year-old woman presented with jaundice, weight loss, abdominal discomfort, and vomiting over 2 months. Laboratory tests showed elevated bilirubin and anemia without infectious signs. Further examinations, including esophagogastroduodenoscopy and abdominal CT scan, revealed duodenal deformation and a pancreatic head mass measuring 6 × 5 × 4 cm, involving the celiac trunk. EUS-guided biopsies confirmed pancreatic ductal adenocarcinoma. Due to the challenging duodenal stenosis, a successful combined EUS-guided gastroenterostomy (EUS-GE) and choledocoduodenostomy was performed [Figure 1]. The patient was discharged on day 3, transitioning from a liquid to a soft diet at home. At the 30-day follow-up, she showed resolution of symptoms.

**Figure 2:**
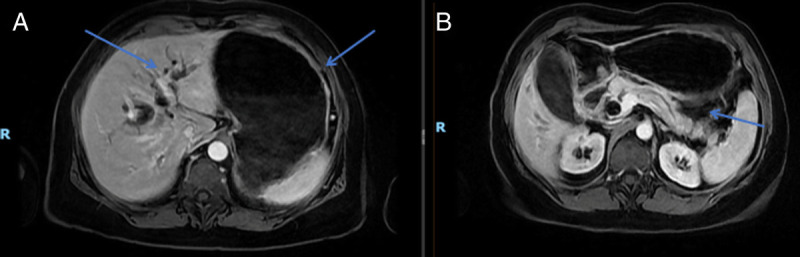
Preprocedural MRI. A, Gastric distention and dilation of intrahepatic bile ducts. B, Dilation of main pancreatic duct. MRI: Magnetic resonance imaging.

**Figure 3:**
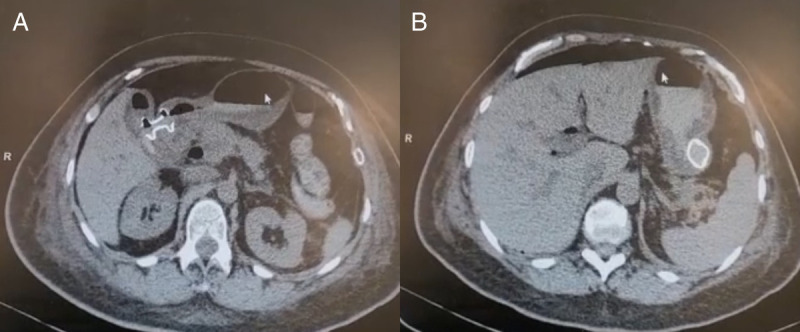
Postprocedural CT scan. A, Tomographic imaging EUS-guided cholecystogastrostomy with LAMS in adequate position. B, Tomographic imaging of EUS-guided gastroenterostomy with LAMS in adequate position. CT: Computed tomography; LAMS: Lumen-apposing metal stent.

In the second case, a 59-year-old woman, previously treated for metastatic breast cancer, developed GOO and jaundice 6 years later. Abdominal magnetic resonance imaging revealed infiltrative tissue in the common bile duct and duodenum, leading to dilation of the stomach and biliary tract [Figure 2]. A double EUS-guided bypass, combining EUS-GE and cholecystogastric EUS-guided anastomosis, was performed due to a more favorable gallbladder disposition [Figure 3]. Postprocedure, she transitioned to a soft diet and was discharged on day 7. At the 90-day follow-up, she maintained a soft diet without obstructive symptoms.

Combined EUS-guided biliary drainage (EUS-BD) and EUS-guided gastroenterostomy (EUS-GE) in a single session is an advanced approach for symptom relief in palliative patients with advanced oncologic diseases, and the initial performance of EUS-BD does not seem to increase the risks for subsequent EUS-GE. The advent of cautery-enhanced lumen-apposing metal stent (LAMS) streamlines the procedure and enhances reproducibility, allowing a safe freehand approach with high success rates when basic principles of therapeutic echoendoscopy and EUS-guided drainages are followed.
